# Linking Diversity and Mental Health: Task Conflict Mediates Between Perceived Subgroups and Emotional Exhaustion

**DOI:** 10.3389/fpsyg.2020.01245

**Published:** 2020-06-30

**Authors:** Niklas Schulte, Friedrich M. Götz, Fabienne Partsch, Tim Goldmann, Lea Smidt, Bertolt Meyer

**Affiliations:** ^1^Department of Psychology and Education, University of Ulm, Ulm, Germany; ^2^Department of Psychology, University of Münster, Münster, Germany; ^3^Department of Psychology, University of Cambridge, Cambridge, United Kingdom; ^4^Department of Psychology, University of Mannheim, Mannheim, Germany; ^5^Department of Economics and Business Administration, Goethe University Frankfurt, Frankfurt, Germany; ^6^Cologne Center for Comparative Politics, University of Cologne, Cologne, Germany; ^7^Department of Psychology, Chemnitz University of Technology, Chemnitz, Germany

**Keywords:** diversity, faultlines, subgroups, conflict, strain, stress, emotional exhaustion

## Abstract

Diversity and psychological health issues at the workplace are pressing issues in today’s organizations. However, research linking two fields is scant. To bridge this gap, drawing from team faultline research, social categorization theory, and the job-demands resources model, we propose that perceiving one’s team as fragmented into subgroups increases strain. We further argue that this relationship is mediated by task conflict and relationship conflict and that it is moderated by psychological empowerment and task interdependence. Multilevel structural equation models on a two-wave sample consisting of 536 participants from 107 work teams across various industries and work contexts partially supported the hypotheses: task conflict did indeed mediate the positive relationships between perceived subgroups and emotional exhaustion while relationship conflict did not; effects on stress symptoms were absent. Moreover, contrary to our expectations, neither empowerment, nor task interdependence moderated the mediation. Results indicate that team diversity can constitute a job demand that can affect psychological health. Focusing on the mediating role of task conflict, we offer a preliminary process model to guide future research at the crossroads of diversity and psychological health at work.

## Introduction

Modern organizations rely on teams that are becoming increasingly diverse due to demographic changes, migration, and other factors (Süß and Kleiner, 2007; Humes et al., 2011; Frey, 2015). Along with ethnicity diversity, further types of (demographic) diversity like gender and age influence group dynamics and work group outcomes (e.g., Joshi and Roh, 2009; Berge et al., 2016). While prior research has often focused on the effects of diversity on performance (for meta-analyses, see for example Bell et al., 2011; van Dijk et al., 2012), little is known about its impact on mental health. However, caring for employees’ emotional and psychological health become increasingly important. For example, while mental health impairments accounted for only 2% of sick leaves in Germany 40 years ago, this figure has grown to 14.7% in 2014 (Knieps and Pfaff, 2015), leading to direct costs of approximately €16 billion for the German economy per year (Bundesanstalt für Arbeitsschutz und Arbeitsmedizin [Federal Agency for Occupational Safety and Health], 2013). The situation is similar in the rest of Europe and in the United States (American Psychological Association Practice Organization, 2010; Eurostat, 2010).

So far, the increasing levels of diversity and the increasing prevalence of psychologic health issues at the workplace are typically in the focus of distinct lines of research. On the one hand, there is little doubt that diversity – at least if distributed in a way that favors the emergence of subgroups within a team – results in conflicts among team members (e.g., Choi and Sy, 2010; Thatcher and Patel, 2012). On the other hand, conflicts constitute a job demand, which, according to the job demands-resources model (Bakker and Demerouti, 2017), can negatively impact employees’ health (e.g., Giebels and Janssen, 2005). The purpose of this study is therefore to integrate these research traditions of diversity and stress. Building on the social categorization theory and the job demands-resources model, we investigate whether and how team diversity can affect employee’s psychological health.

To our best knowledge, the only existing study on a potential link between team diversity and employees’ mental health (Wegge et al., 2008) did not investigate the psychological process behind such an effect and only looked at two diversity attributes (age and gender diversity) without taking team members’ perceptions into account. Therefore, the present study seeks to deepen our understanding of the association between team diversity and mental health in several ways. First, instead of single-attribute diversity indicators, our work draws upon faultline theory as an alternative approach describing how multi-attribute diversity in a team is distributed. Faultlines are hypothetical dividing lines splitting a team into relatively homogeneous subgroups on the basis of multiple attributes (Lau and Murnighan, 1998). Compared to single-attribute diversity measures, faultlines capture not only the heterogeneity of team member characteristics but their distribution, too. Consequently, two teams with the same overall demographic makeup can have different faultline strengths. The faultline construct has been shown to produce less heterogeneous results compared to conventional diversity measures (Thatcher and Patel, 2012).

Second, faultline research distinguishes dormant from active faultlines. Dormant faultlines describe the objective demographic alignment. When team members actually perceive this alignment as the division of the group into subgroups, the faultline is activated (Jehn and Brezrukova, 2010). Most existing faultline studies focus on dormant faultlines and do not account for group members actual perceptions. Active faultlines, in contrast, have been primarily investigated in lab studies so far (Thatcher and Patel, 2012). However, the demographic characteristics themselves are only a proxy of the true psychological mechanisms that can cause negative (or positive) effects for individuals and teams. It is not surprising therefore that the effects of faultlines are especially pronounced if team members subjectively perceive a formation of subgroups within their team (Thatcher and Patel, 2012). This finding is mirrored in the observation that the salience of social categorizations moderates the effect of objective faultlines on team outcomes (Meyer et al., 2011). Put differently, it is not the mere existence of faultlines but rather the emergence of subgroups through categorization processes that unlocks the negative potential of inter-subgroup bias whereby subgroup members favor members of the own subgroup and disadvantage others (see also Carton and Cummings, 2012). Focusing on objective diversity indicators ignores the ambiguity associated with them, as they may or may not lead to categorization processes that ultimately can give rise to conflicts and the associated psychological strain. In contrast, when team members subjectively perceive subgroups, social categorization has already taken place (see also Meyer et al., 2015). By investigating perceived subgroups (i.e., active faultlines), we attempt to address the psychological process directly rather than possible preconditions.

As a third contribution, this study seeks to establish conflicts as the mechanism linking perceived subgroups with mental health as theorized (but not tested) previously (Wegge et al., 2008). In sum, this study addresses the need of western societies to deal with their diversified workforces. It is designed to identify challenges that can be overcome with existing programs (e.g., for stress prevention). This might help accepting diversity in the workforce, which is a critical precondition for making economic use of diversity’s potential.

### Perceived Subgroups and Conflicts

Potential negative effects of team diversity are generally explained with social categorization theories (see van Knippenberg and Schippers, 2007, for a review), whereby individuals categorize themselves and others into ingroup and outgroup members based on perceived similarities and differences among team members (Williams and O’Reilly, 1998). If a subset of team members aligns along multiple attributes such as gender, age, or personal values, those team members will likely form a homogeneous subgroup resulting in social differentiation processes (Lau and Murnighan, 1998; Meyer et al., 2014). These processes lead to adverse outcomes through intergroup bias, whereby members of the ingroup are perceived more favorably than members of the outgroup (Tajfel et al., 1971; van Knippenberg and Schippers, 2007).

Social categorization is associated with lower levels of trust, a reduced willingness to cooperate and subsequently more competition with members of the outgroup (Brewer, 1979; Kramer and Brewer, 1984; Tajfel and Turner, 1986; Brewer and Brown, 1998). Social categorization processes among the members of the same work unit, therefore have been associated with a host of negative outcomes, including reduced team performance, lower team cohesion, and increased turnover (O’Reilly et al., 1989; Murnighan and Conlon, 1991). Moreover, according to the categorization-elaboration model (van Knippenberg et al., 2004), diversity-driven social categorization processes can lead to conflicts among the members of the respective (sub-) groups. In line with these findings, active faultlines (and thus perceived subgroups) are likely to increase team conflicts (Jehn and Bezrukova, 2010; Thatcher and Patel, 2012).

Team conflicts at the workplace can be categorized into three types: relationship conflict, task conflict, and process conflict (Jehn and Mannix, 2001; O’Neill et al., 2013; but see Bendersky et al., 2010, as well as Bendersky and Hays, 2012, for the discussion of status conflict as a potential fourth facet). Relationship conflict refers to interpersonal incompatibilities involving a strong affective component (Jehn, 1995, 1997; Jehn and Mannix, 2001). Task conflict in turn concerns disagreements over the task at hand such as its content and goals (Jehn, 1995, 1997; Jehn and Mannix, 2001). Finally, process conflict arises over how the work is done, e.g., regarding duty and research delegation (Jehn, 1997; Jehn and Mannix, 2001).

Theories linking perceived subgroups and relationship conflict build on the social categorization processes described earlier (Pelled et al., 1999; van Knippenberg and Schippers, 2007). The categorization into distinct subgroups can provoke hostility or animosity between these subgroups (Jehn et al., 1999). This hostility can surface as gossip, exclusion from social events or other behaviors that are harmful for social relations (Jehn, 1995, 1997). The close contact with members of the same homogeneous subgroup facilitates this processes as each team member can assume a common aversion against dissimilar others and thus engage in these harmful behaviors more carelessly compared to groups in which animosities are less obviously distributed. Therefore, intergroup bias resulting from social categorization processes can be a direct cause of relationship conflict (Hornsey and Hogg, 2000; Pickett and Brewer, 2001; van Knippenberg and Schippers, 2007; Jehn and Bezrukova, 2010; Thatcher and Patel, 2012; Hentschel et al., 2013).

Similar evidence exists for the relationship between perceived subgroups and task conflict (Thatcher and Patel, 2012): a group characterized by homogeneous subgroups will usually harbor a greater probability of differing views regarding the task at hand, constituting a potential source of conflict (Pelled et al., 1999). Usually, team members align their viewpoints among each other to a certain degree to satisfy affiliation needs or epistemic motives in ambiguous situations (Echterhoff et al., 2009). It is thus more likely that these processes are especially pronounced within a homogeneous subgroup. Additionally, the increased within-subgroup and decreased inter-subgroup exchanges that characterize subgroups formed by (active) faultlines (Lau and Murnighan, 2005) create more occasions for members of the same subgroup to share opinions and create a commonality with each other. Shared perspectives facilitate the formations of subgroups and are likely to further polarize over time due to the above-described processes. In sum, in line with subgroup theory (Carton and Cummings, 2012), different subgroups are likely to develop different viewpoints, which are likely to result in dissent and task conflict (van Knippenberg et al., 2004). In sum, our arguments suggest that subgroups will not only cause relationship conflict, but also task conflict. To avoid adding complexity to our study, we chose to not include process conflict in our research. A meta-analysis (de Wit et al., 2012) also shows that process conflict correlates highly (ρ = 0.72) with task conflict and relationship conflict (ρ = 0.72). Although process conflict evokes own effects, they are often in line with the other conflict facets. Like many primary studies in the above mentioned meta-analysis, we did not include process conflict in the following for parsimony but expect a similar picture for this conflict facet.

### From Conflicts to Strain

Conflicts are not only a consequence of social categorization processes (c.f., categorization-elaboration model) but can also be regarded as an input variable in stress models: the Job-Demand-Resources (JD-R) model categorizes workplace characteristics as either demands or resources. A job demand is any aspect of the workplace, be it organizational, physical, psychological, or social, that accrues physiological and psychological costs. These can include quantitative workload but also distressing interactions with clients and/or colleagues (Spector and Jex, 1998). Job resources on the other hand refer to factors that facilitate reaching work goals, dealing with job demands, and/or to factors that stimulate personal development. These can range from work autonomy to belief in personal skills and abilities (Demerouti et al., 2001). In linking social categorization theory to the JD-R, we position conflicts resulting from subgroup perception as a work condition that acts as a potential job demand (Keenan and Newton, 1985; Spector and Jex, 1998; Giebels and Janssen, 2005). Conflicts are therefore likely to affect employee health, i.e., cause stress or strain.

However, when it comes to stress, the literature uses an inconsistent terminology and an array of contradicting definitions. Stress can be conceptualized in different ways, e.g., as a stimulus, as a response, or as a transactional concept (Cooper et al., 2001). We refer to stress as a process that can result in different manifestations (Pearlin et al., 1981) as we explain in the following. To avoid ambiguity, we separate job demands from strain with the latter being the potential result of a prolonged exposure to job demands. The sequence wherein stressors evoke strain is labeled as *stress process* here (Sutherland and Cooper, 1990). Individuals can be affected by the stress process in multiple ways, i.e., by showing strain symptoms on the physiological, on the psychological, and on the behavioral level (Schuler, 1980; Sutherland and Cooper, 1990). This study focuses on two strain manifestations, namely emotional exhaustion and behavioral stress symptoms, which we introduce in the following.

According to the JD-R, job demands can lead to a state of exhaustion and health problems if individuals have no proper resources at their command. The concrete dependent variable in the JD-R has changed over time (Demerouti et al., 2001; Bakker and Demerouti, 2007, 2017). The initial model (Demerouti et al., 2001) focused on burnout. Burnout encompasses the facets: emotional exhaustion, depersonalization, and feelings of reduced personal accomplishment (Maslach and Jackson, 1981; Maslach, 2003). Within the burnout facets, emotional exhaustion is the facet that is most closely tied to the health realm (Bakker and Demerouti, 2017). It is “at the heart of the burnout syndrome” (Maslach, 2003, p. 3) and represents the most immediate result of the stress process among the three burnout components (Maslach et al., 2001). As this study seeks to investigate the direct health effects of group dynamics, we will focus on emotional exhaustion as the core health facet of burnout.

Integrating new empirical findings, later versions of the JD-R replaced burnout with strain as the outcome of the stress process (Bakker and Demerouti, 2007, 2017) and in the latest version, strain is used as an umbrella term for all kinds of health impairments (Bakker and Demerouti, 2017). Following this holistic approach to cover different aspects of the human stress response, we investigate behavioral stress symptoms as a second manifestation of strain. We argue that individuals who are faced with excessive job demands show behavioral changes like withdrawal and a lack of drive. For reasons of simplicity, we use the term stress when referring to behavioral stress symptoms in the following. By capturing symptoms from two different categories of strain manifestations (i.e., psychological and behavioral aspects), we aim at a broader and more comprehensive representation of strain than the exclusive focus of burnout or one of its facets would allow.

Having established both the link between perceived subgroups and conflict according to social categorization theory as well as the relation between conflict and strain based on the JD-R model, we propose:

*Hypothesis* 1: Perceived subgroups are positively related to strain.
*Hypothesis* 2a: The relationship between perceived subgroups and strain is mediated by relationship conflict.
*Hypothesis* 2b: The relationship between perceived subgroups and strain is mediated by task conflict.

### Task Interdependence as a Moderator of the Perceived Subgroup-Conflict Relationship

Above we have argued that perceived subgroups cause strain *via* conflict. We now turn to two potential moderators of this relationship, namely task interdependence and psychological empowerment.

We posit task interdependence – the degree to which team members depend on one another to complete a given task (Campion et al., 1993) – as a potential moderator of the relationship between perceived subgroups and conflict. According to the categorization elaboration model, the nature of the task moderates the connection between diversity and work outcomes. With regard to the task interdependence, it suggests that diversity should have a particularly strong impact when the task requires cooperation. Task interdependence constitutes a structural aspect of the task environment that forces team members to interact in order to achieve team goals. In a team that is split into subgroups, team members will favor members of their own subgroup (Tajfel et al., 1971) minimizing the interaction with discriminated others. In such a situation, different viewpoints and reservations will exist on a latent level. When people with latent animosities have to interact due to interdependencies regarding their work task, these tensions will surface as manifest conflicts. In sum, we propose:

*Hypothesis* 3a: Task interdependence moderates the relationship between perceived subgroups and relationship conflict. That is, when team members perceive strong subgroups, teams with high task interdependence will experience higher levels of relationship conflict compared to teams with low task interdependence.
*Hypothesis* 3b: Task interdependence moderates the relationship between perceived subgroups and task conflict. That is, when team members perceive strong subgroups, teams with high task interdependence will experience higher levels of task conflict compared to teams with low task interdependence.

### Psychological Empowerment as a Moderator of the Conflict-Strain Relationship

While task interdependence is a moderator within the social categorization framework, we derive psychological empowerment as a moderator of the relationship between conflict and strain from the JD-R framework. Psychological empowerment is a core cognition toward work and is a higher-order construct defined by the Gestalt of the four cognitions meaning, competence, self-determination, and impact (Spreitzer, 1995; Spreitzer et al., 1997). Meaning relates to the alignment of one’s personal values with the purpose of the work role. Impact describes the degree to which an individual can influence the outcomes at work. Competence on the other hand is the belief in one’s ability to have the necessary skills to deal with the task at hand. Self-determination encompasses the degree to which individuals are able to shape their own work processes. Empowerment derives from people’s perception of their work environment and is thus subject to change, rather than being an enduring personality trait (Spreitzer, 1995).

In line with the JD-R, psychological empowerment acts as a buffer against demands experienced in the workplace (Schermuly and Meyer, 2015) and is in turn correlated with lower levels of strain (Seibert et al., 2011). Empowerment also reduces emotional exhaustion specifically (*via* increased job satisfaction; Schermuly et al., 2011). As explained above, we posit conflict as an important stressor in the JD-R context. In line with prior studies (Schermuly and Meyer, 2015), we expect psychological empowerment to act as a buffering resource on this demand-strain relationship. In particular, job autonomy, a sense of being in control of one’s work context (conceptually very close to empowerment) can be an important resource for employees by providing flexibility in dealing with job demands (Bakker and Demerouti, 2007). This is corroborated by studies showing that autonomy can buffer negative effects of job demands on exhaustion and cynicism (Bakker et al., 2005). In further support of this hypothesis, psychological empowerment increases an individual’s ability to deal with stressors (Pines et al., 2011; Thomas and Revell, 2016). In addition, the self-efficacy facet of empowerment can be an important tool in managing conflict situations and hence help attenuating its negative consequences (Jex and Bliese, 1999) and has been modeled as a resource in past stress research (Bakker and Demerouti, 2017). We therefore propose:

*Hypothesis* 4a: Psychological empowerment moderates the relationship between relationship conflict and strain. That is, the impact of conflicts of team members’ strain level is weaker, when empowerment is high.
*Hypothesis* 4b: Psychological empowerment moderates the relationship between task conflict and strain. That is, the impact of conflicts on team members’ strain level is weaker, when empowerment is high.

## Methods

### Sample

We conducted a two-wave time-lagged field study. The sample consists of teams from various German organizations and occupations, such as police officers, office workers, and members of association committees. Overall, 536 participants from 107 teams participated in the survey at least one point in time. Of these, 470 participants from 106 teams with an average size of *M* = 6.00 members (*SD* = 3.14). A total of 178 team members from 58 teams with an average size of *M* = 4.34 members (*SD* = 2.29) completed the follow-up questionnaire. The response rate dropped from 73% at t_1_ to 28% at t_2_, which is commensurate with typical attrition rates in organizational psychology (e.g., Griffin et al., 2010; Schermuly and Meyer, 2015). The sample collected in the first wave included 58% women. The average age of all respondents was 34.36 years (*SD* = 11.92) ranging from 16 to 68. Of the respondents at t_1_, 328 (71%) had a professional qualification and 136 (29%) occupied a leading position within their respective team. In the second wave, 111 (63%) of the respondents were female, 92 (83%) had a professional qualification and ages range between 19 and 68 with an average of *M* = 38.23 years (*SD* = 11.45). The number of team leaders dropped to 41 (23%) among the respondents in the second wave.

The teams were recruited by participants of a summer school who advertised the study in their wider network in various professional and non-professional work-related contexts. Participants did not receive any material incentive but team-specific feedback and recommendations for improving team collaboration. This study was part of a wider data collection effort covering a variety of constructs relevant for diversity studies in team contexts. A data transparency table is available from the authors.

### Design

The present study is a questionnaire study with two times of measurement. Respondents filled in two questionnaires at their work place. Even though longitudinal studies regarding the JD-R were recently analyzed meta-analytically, the ideal time interval to prove causal effects of job characteristics on strain remains unclear (Lesener et al., 2018). We decided for a 3-month time lag. This interval appeared long enough to avoid artifactual covariance of our predictor and criterion variables (Podsakoff et al., 2003) and allows the study variables to vary. But still, the interval is short enough to avoid serious sample attrition (Daniel and Sonnentag, 2014) and limits the influence of staffing decisions on team configurations. While the first questionnaire required approximately 30 min, the length of the follow-up questionnaire decreased to 15 min. We report data for independent, mediator, and moderator variables at t_1_ and dependent strain variables at t_2_. Thus, the study is cross-sectional for the associations between independent and mediator variables but allows a prediction of strain from t_1_ to t_2_.

### Measures

#### Perceived Subgroups

Lacking an established measurement instrument in German, we developed a scale for operationalizing perceived subgroups, see Appendix, based on similar English short scales (Zellmer-Bruhn et al., 2008; Homan and Greer, 2013). We measured perceived subgroups with a seven-point Likert scale ranging from *strongly disagree* to *strongly agree* on the three items “Members of our team who are similar to each other interact more frequently,” “In my team, the same groups of people discuss among themselves,” and “Within my team, different subgroups have emerged whose members get along very well.” The internal consistency of the scale was *α* = 0.78.

#### Strain

As discussed above, we operationalized the dependent variable strain in terms of stress and emotional exhaustion. We did not match stress and emotional exhaustion for an overall score, but hypothesized effects to be similar on both outcome variables. To measure stress, we used the German translation (Nübling et al., 2005) of the COPSOQ (Kristensen et al., 2005; Pejtersen et al., 2010). The scale measures behavioral stress symptoms with six items (e.g., *I had difficulties to feel happy*; Cronbach’s *α* = 0.84). To measure emotional exhaustion we used five items (e.g., *I feel used up at the end of the workday*) of the German Maslach Burnout Inventory seven items subscale (MBID; Büssing and Perrar, 1992, 1994), which exhibited an internal consistency of *α* = 0.90.

#### Intragroup Conflict

We used the German version of Jehn’s (1995) scale to measure two facets of intragroup conflict, namely task conflict (e.g., *How often do people in your work unit disagree about opinions regarding the work being done?*) and relationship conflict (e.g., *How much tension is there among members in your work unit*; Lehmann-Willenbrock et al., 2011). The internal consistency of the three item scales are *α* = 0.79 for relationship conflict and *α* = 0.81 for task conflict.

#### Psychological Empowerment

We used the German version of Spreitzer’s (1995) 12 item scale for measuring psychological empowerment (Adolf et al., 2009). It contains three items for each of the construct’s four dimensions (meaning, competence, self-determination, and impact), resulting in an overall empowerment score by averaging the scores of the items. The overall alpha for empowerment in our sample was *α* = 0.86.

#### Task Interdependence

We used the German version of van der Vegt’s and Janssen’s (2003) scale to measure task interdependence. The scale encompasses five items (e.g., *To finish my work I need information and tips from my team colleagues*.) resulting in an internal consistency of *α* = 0.75 at t_1_.

In addition to the main independent variables, we controlled for age and gender at t_1_ and t_2_.

## Results

### Analysis Strategy

We calculated descriptive statistics calculated in R (R Core Team, 2018) with the packages psych (Revelle, 2016), sjPlot (Lüdecke, 2017), multilevel (Bliese, 2016), and lme4 (Bates et al., 2015). We specified multilevel structural equation models (SEMs) with perceived subgroups, task interdependence, task and relationship conflict, and empowerment at t_1_ and stress as well as emotional exhaustion at t_2_. To rigorously test the model’s ability to predict strain over time, we controlled for stress and emotional exhaustion (i.e., strain) at t_1_ in all our models. Additionally, we controlled for the demographic variables age and gender (each at t_2_) and team size at t_1_. For reasons of parsimony, we removed demographic variables in later models if they showed no significant associations in the model estimated before. Multilevel structural equation models were fitted with Mplus Version 6.11 (Muthén and Muthén, 2011) using the maximum likelihood estimator.

We started our analysis by investigating the hierarchical structure of the data and compared the fit of several SEMs to select the model which fitted our data best. Subsequently, we tested our hypotheses based on the coefficients of the selected model. Regression coefficients reported here are standardized path coefficients for direct and moderating effects and unstandardized path coefficients for indirect effects, respectively.

### Model Selection

The data has two levels: individuals are nested in teams. To test for potential non-independence caused by the hierarchical structure of the data, we followed recommendations by Bliese (2002, 2016) and calculated intra-class-correlations (ICCs; Shrout and Fleiss, 1979) for all dependent and mediating variables. Both stress, ICC(1) = 0.00, *F*(56, 116) = 0.94, *p* = 0.60, ICC(2) = 0.00, and emotional exhaustion, ICC(1) = 0.06, *F*(56, 110) = 1.17, *p* = 0.24, and ICC(2) = 0.15, did not depend on the membership in a certain team. Regarding the mediators, a significant amount of variance was explained by team membership, both for task conflict, ICC(1) = 0.39, *F*(104, 343) = 3.70, *p* < 0.001, and ICC(2) = 0.73, and relationship conflict, ICC(1) = 0.47, *F*(104, 343) = 4.83, *p* < 0.001, and ICC(2) = 0.79. We, thus, tested the hypotheses with mixed (i.e., multilevel) path models.

To explore the random effect structure, we compared random-intercept with random intercept-random-slope models for the focal predictors and perceived subgroups as the independent variable with Chi-square difference tests based on the difference in model deviance (Bliese, 2016). Consistent with the ICC patterns, a random-intercept-and-random-slope model did not fit the data significantly better than a random-intercept model, neither for stress, Δχ^2^(2) = 0.24, *p* = 0.89, nor for emotional exhaustion, Δχ^2^(2) = 2.37, *p* = 0.31. We thus tested the hypotheses with random intercept models.

Testing model assumptions revealed that stress resembled a poisson or negative binomial distribution. However, model diagnostics (e.g., residual Q-Q plots) of a random-intercept model for normally-distributed dependent variables did not indicate a violation of model assumptions (i.e., residuals were normally distributed). Moreover, neither a generalized model with a poisson link function, nor a generalized model with a negative binomial link function resulted in better fits. Taken together, the assumptions of the initial model were not violated and we therefore employed a random-intercept model for normally-distributed dependent variables to test our hypotheses.

### Model Specification

Means, standard deviations, intra-class and bivariate correlations for all study variables are reported in Table 1. To test the proposed model, we estimated path models in the structural equation framework. In a first step, we tested the mediation of the relationship between perceived subgroups and stress as well as emotional exhaustion *via* relationship and task conflict while controlling for age, gender, and team size in all other variables. Additionally, we controlled for stress at t_1_ in the prediction of stress at t_2_ and stress and emotional exhaustion (both at t_1_) in the prediction of emotional exhaustion at t_2_.

**Table 1:** Means, standard deviations, intra-class-correlations (ICCs), and bivariate correlations for all study variables.

Measure	*M*	*SD*	ICC1	ICC2	1	2	3	4	5	6	7	8	9	10	11
1. Perceived subgroups	4.25	1.39	0.20	0.51											
2. Task conflict	2.81	0.72	0.39	0.73	0.24^***^										
3. Relationship conflict	2.31	0.76	0.47	0.79	0.33^***^	0.55^***^									
4. Task interdependence	4.48	1.20	0.15	0.43	−0.09*^†^*	−0.03	−0.11^*^								
5. Empowerment	5.65	0.82	0.09	0.28	−0.19^***^	−0.18^***^	−0.19^***^	0.14^**^							
6. Stress t_1_	1.68	0.68	0.07	0.24	0.33^***^	0.28^***^	0.40^***^	−0.18^***^	−0.39^***^						
7. Stress t_2_	1.66	0.63	0.00	0.00	0.22^*^	0.20^*^	0.39^***^	−0.11	−0.34^***^	0.72^***^					
8. Emotional exhaustion t_1_	2.67	1.05	0.12	0.36	0.15^**^	0.15^**^	0.30^***^	−0.02	−0.35^***^	0.05^***^	0.55^***^				
9. Emotional exhaustion t_2_	2.76	1.07	0.06	0.15	0.08	0.12	0.22^*^	0.00	−0.33^***^	0.46^***^	0.65^***^	0.75^***^			
10. Age	38.23	11.45	0.43	0.62	−0.04	−0.12	0.20^*^	−0.04	0.15	−0.06	−0.15*^†^*	0.02	−0.13*^†^*		
11. Gender			0.22	0.39	0.03	−0.05	0.06	0.00	−0.07	−0.01	0.02	−0.07	−0.04	0.00	
12. Team size	10.57	8.49	0.84	0.96	0.15^**^	0.21^***^	0.02	0.12^*^	0.01	0.00	0.13	−0.06	0.17*^†^*	−0.08	−0.07

*N* = 167-460, ICC = intra-class-correlations; t_1_ = the first measurement; t_2_ = the second measurement.

† *p* < 0.10;

* *p* < 0.05;

** *p* < 0.01;

*** *p* < 0.001.

Of all demographic variables, only age at t_2_ showed a significant effect on stress, *b* = −0.15, *p* = 0.02, and a marginally significant effect on emotional exhaustion, *b* = −0.12, *p* = 0.08. For reasons of model parsimony, we dropped the other non-significant demographic variables.

In a third step, we added the moderator’s *task interdependence* and *empowerment* into the model (Model 3). In this model, we controlled for age and stress at t_1_, and emotional exhaustion at t_1_ in all study variables and interaction terms. When testing the potential moderators, we controlled for the correlation between the three relevant variables associated with the moderating effect as well as for the correlation of the associated variables and their interaction terms. Additionally, we specified correlations between perceived subgroups, both moderators, and their interaction terms. Fit indices of the three structural equation models are shown in Table 2.

**Table 2:** Fit indices for structural equation models.

Model	χ^2^	*df*	χ^2^/*df*	RMSEA	SRMR	TLI	CFI
Model 1	112.03	48	2.33	0.05	0.04	0.82	0.90
Model 2	86.55	28	3.09	0.06	0.03	0.82	0.91
Model 3	168.20	88	1.91	0.04	0.04	0.87	0.93

RMSEA = root mean square error of approximation; SRMR = standardized root mean square residual on within level; TLI = Tucker-Lewis index; CFI = comparative fit index. Model 1 includes all controls, Model 2 controls for age at t_2_, and Model 3 contains all moderator variables while controlling for age at t_2_.

According to the criteria laid out by Schermelleh-Engel et al. (2003), the goodness-of-fit indices for Model 1 (all controls, no moderators) indicated a good fit based on the RMSEA and the SRMR, an acceptable fit with respect to the χ^2^/*df* ratio, but a non-acceptable fit based on the TLI and CFI (see Table 2). After removing the insignificant control variables gender and team size (Model 2), the SRMR reached good fit, the RMSEA indicated an acceptable fit, whereas the χ^2^/*df* ratio as well as the relative fit indices TLI and CFI did not indicate an acceptable fit. Model 3 yielded a good fit based on the χ^2^/*df* ratio, the RMSEA and the SRMR but no acceptable fit based on the TLI and the CFI.

**Figure 1:**
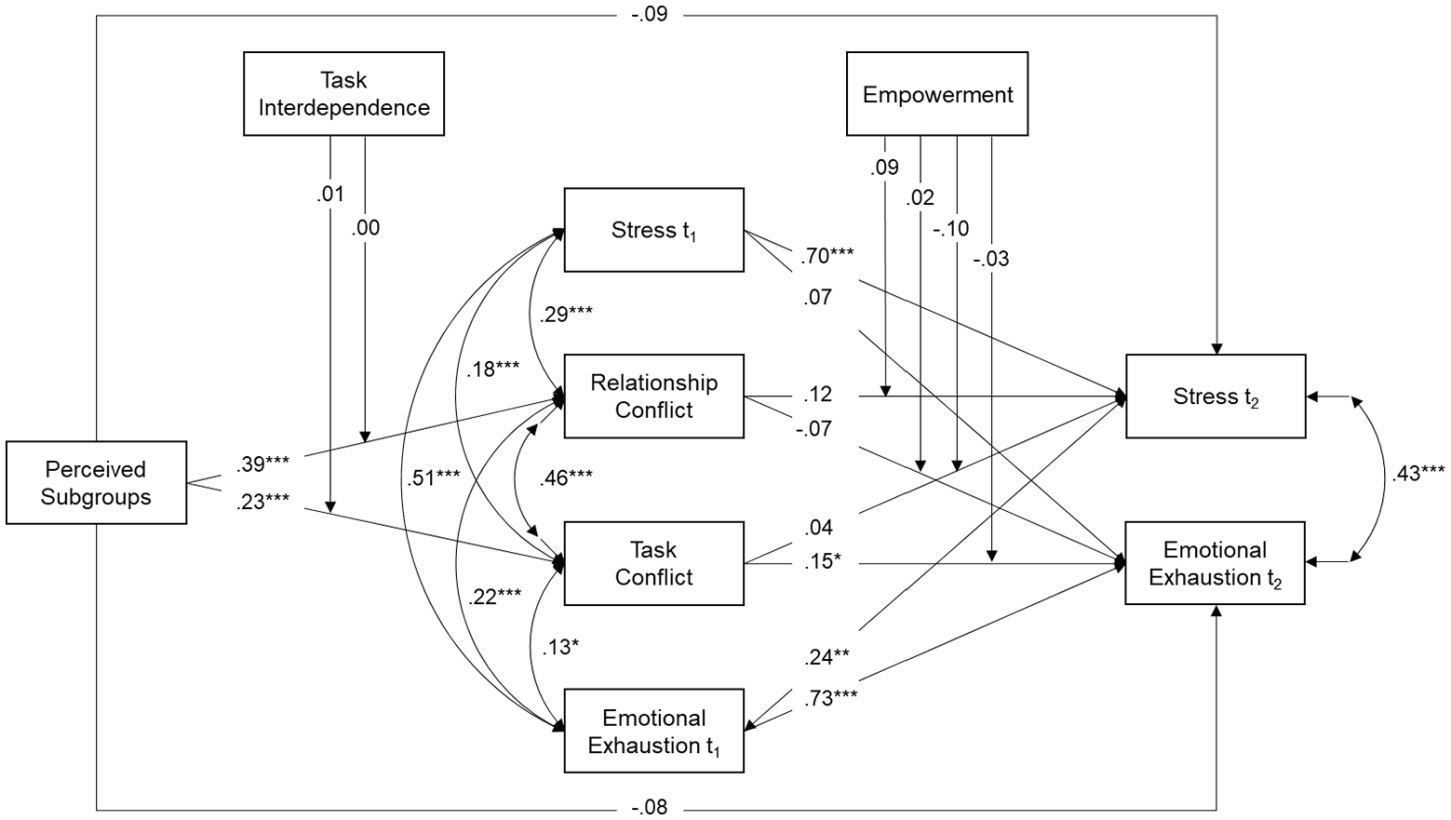
Standardized model results for Model 3. *N* ꞊ 515 (Level 1) and *N* ꞊ 105 (Level 2). We controlled for age at t_2_ in all study variables. Note that we controlled for age, stress at t_1_, and emotional exhaustion at t_1_ in all study variables and interaction terms. We also controlled for the correlation between three relevant variables associated with the moderating effect as well as for the correlation of the associated variables and their interaction terms. Additionally, we specified correlations between perceived subgroups, both moderators, and their interaction terms. Amounts of variance explained are R^2^ ꞊ 0.18 for relationship conflict, R^2^ ꞊ 0.14 for task conflict, R^2^ ꞊ 0.62 for stress, and R^2^ ꞊ 0.66 for emotional exhaustion. ^*^p < 0.05, ^**^p < 0.01, ^***^p < 0.001 (two-tailed).

Of the different models tested here, Model 3 exhibited the best fit. Model 3 and its standardized path coefficients are presented in Figure 1. The data as well as the analysis and result scripts for the structural equation models are available in the OSF^1^.

### Hypotheses Testing

We now report results for hypotheses tests that are based on the path coefficients of Model 3. We used two-tailed tests for regular path coefficients and we employed one-tailed tests for the mediation paths. This was justified by the low power that tests for mediation effects usually have (Hayes, 2013) combined with theoretical considerations (i.e., we expected positive effects).

Our first hypothesis was concerned with the effect of perceived subgroups on strain. Hypothesis 2a assumed that this effect is mediated by relationship conflict, while Hypothesis 2b suggested mediation *via* task conflict. We found no evidence for an effect of perceived subgroups on stress, neither direct, *b* = −0.09, *ns*, nor indirect *via* relationship conflict, *b* = 0.05, *ns*, or task conflict, *b* = 0.01, *ns*. However, perceived subgroups exhibited an indirect effect on emotional exhaustion *via* task conflict, *b* = 0.04 and *p* = 0.03. In contrast, perceived subgroups were neither directly associated with emotional exhaustion, *b* = −0.08, *ns*, nor was this association mediated by relationship conflict, *b* = −0.03, *ns*. Taken together, these results indicate that individuals who perceive their team as fragmented into subgroups experience more task conflict which in turn leads to higher levels of emotional exhaustion. Hypotheses 1 (association of subgroups and strain) and 2b (mediation *via* task conflict) are therefore supported for emotional exhaustion but not for stress. The results do not support Hypothesis 2a. However, the perception of subgroups was associated with relationship conflict (see Figure 1).

We now turn to the moderation tests. As shown in Figure 1, task interdependence neither moderated the influence of perceived subgroups on relationship conflict, *b* = 0.00, *ns*, nor on task conflict, *b* = 0.01, *ns*. Empowerment did not moderate the relationship between relationship conflict and strain (*b* = 0.09, *ns*, for stress, *b* = 0.02, *ns*, for emotional exhaustion). Likewise, empowerment exhibited no significant influence on the influence of task conflict on emotional strain (*b* = −0.10, *ns*, for stress, *b* = −0.03, *ns*, for emotional exhaustion). In line with these results, Hypotheses 3 and 4 had to be rejected.

## Discussion

The present study set out to link perceptions of subgroups to stress and emotional exhaustion, which we conceptualized as manifestations of strain and henceforth mental health issues. With respect to Hypothesis 1, neither stress, nor emotional exhaustion showed direct associations with perceived subgroups when controlling for other variables in our model. However, a more nuanced picture emerged when we ran mediation analyses in keeping with Hypotheses 2a and 2b, respectively. Whereas task conflict did not mediate the relationship between perceived subgroup and stress, it fully mediated the relationship between perceived subgroups and emotional exhaustion. These findings offer partial support for Hypotheses 1 and 2b. Meanwhile, we had to reject Hypothesis 2a, stating that relationship conflict mediates the link between perceived subgroups and strain. Neither task interdependence (Hypotheses 3a and 3b) nor psychological empowerment (Hypotheses 4a and 4b) showed moderating effects.

Interestingly, perceived subgroups were associated with both task and relationship conflict, supporting the part of our model that had not been researched before. In contrast, the theoretically and empirically well-established link between stressors and strain (i.e., both conflict types and both strain variables) was absent in three of four cases in our study. The wide range of industries and occupations covered by our sample make these two potential situational moderators unlikely to be responsible for the limited effects of job demands (i.e., conflicts) on strain in this study. Our findings ought to be interpreted against the backdrop of a comprehensive amount of literature demonstrating the stressor-strain link. While our results do not raise questions about this well-established association *per se*, they indicate limited robustness of these effects in longitudinal studies when controlling for strain levels at t_1_.

Closely linked with the aforementioned observation, the effect discrepancy between the two conflict types appears to be puzzling at first. Their differential impact may be rooted in qualitative distinctions as captured by the challenge-hindrance stressor framework (Cavanaugh et al., 2000; Boswell et al., 2004; LePine et al., 2005). While relationship conflicts constitute a hindrance stressor (Sonnentag and Frese, 2012), scholars have argued that task conflict constitutes a challenge stressor (De Dreu and Weingart, 2003). Unlike hindrance stressors, challenge stressors harbor the potential for growth and learning as positive results of successfully dealing with the stressor in question. Accordingly, in addition to producing strain, challenge stressors boost motivation (LePine et al., 2004; Kubicek and Korunka, 2015). Driven by the prospect of growth, learning, and thriving through successful mastery of the challenge at hand (Kahn, 1990), challenge stressors promote a problem-focused coping strategy (Crawford et al., 2010). Among other things, such coping encompasses continued mental occupation with one’s job (Sonnentag et al., 2010), heightened work engagement (Crawford et al., 2010; Karatepe et al., 2014), job involvement (Yao et al., 2015), dedication (Sonnentag et al., 2010), lower likelihood of withdrawal (Boswell et al., 2004), enhanced willingness to increase one’s efforts (Hockey, 1997; Pelled et al., 1999; Bakker et al., 2014), and commitment (Podsakoff et al., 2007). Looking through this lens, challenge stressors might in fact increase individuals’ vulnerability to strain and burnout, by paving the way for over-commitment (Webster et al., 2011; Widmer et al., 2012). Indeed, challenge stressors may cause employees to go above and beyond, despite feeling exhausted and worn-out (LePine et al., 2005; Webster et al., 2011), which becomes more salient once the energizing effects of challenge stressors wear off (Prem et al., 2017). In our model, we controlled for strain occurring immediately in the presence of the stressor by including both strain variables at t_1_ as predictors for strain at t_2_. Under these conditions, we see the described detrimental long-term effects exclusively for challenge stressors. In this vein, our results offer further empirical support for the idea that harmful effects of (challenge) stressors may under some circumstances not develop immediately but rather gradually over time and may thus only become visible in the long run (Crawford et al., 2010; for a review and meta-analysis see Ford et al., 2014). This being said, the cross-sectional designs of the majority of studies in the literature may turn out to be a major drawback, obscuring critical effects (Widmer et al., 2012; Rispens and Demerouti, 2016). Indeed, the few existing longitudinal studies in this line of research provide strong empirical support for the assumed time-lagged nature of the aforementioned effects (de Lange et al., 2003; Hakanen et al., 2008).

Furthermore, results reveal a more differentiated picture not only for stressors but also for strain outcomes. While we successfully demonstrated effects of task conflict on emotional exhaustion, we found no association with stress. The reason for this pattern of results might again be the development of these effects over time. Stress symptoms measured here are conceptualized as a rather immediate response to stressors while emotional exhaustion constitutes a long-term ramification of chronically elevated exposure to stressors. In light of the present study’s results, we assume that stress symptoms caused by stressors at t_1_ have vanished after several months of time lag. Stress levels which we see at t_2_ might not be caused by exposure to stressors (e.g., task conflict) at t_1_ but stressors occurring immediately before t_2_. Because we controlled for stress at the first measurement point, the time-stable elements of this measure were not considered in our analysis and did therefore not contribute to the prediction of stress at t_2_.

With respect to relationship conflict, we did not detect any consequences for strain levels when controlling for strain levels at t_2_. We assume that the time lag of several months between both measurement points was too long to show effects. As mentioned above, hindrance stressors have been demonstrated to promote avoidant coping strategies (Jehn, 1995; Pearsall et al., 2009; O’Neill et al., 2013). While this is clearly detrimental to performance outcomes, it may actually be rather adaptive and protective on an individual level. Our results suggest that this might be especially true in the long run. Preventing over-commitment and facilitating relaxation and recovery (Sonnentag and Bayer, 2005; Sonnentag et al., 2010), psychological detachment and mentally disconnecting from work appear to shield against emotional exhaustion (Sonnentag et al., 2010; Rispens and Demerouti, 2016; Schneider et al., 2017). Taken together, the present study hints at a more complex interplay of challenge and hindrance stressors with strain than has been assumed in prior research, with the research context of team member diversity adding a further layer of complexity.

Moving on to Hypothesis 3, none of the corresponding path coefficients for the postulated interactions of task interdependence with perceived subgroups and conflict indicated a significant influence of the interaction terms. The lack of moderating effects could be the result of counteracting influences, ultimately neutralizing each other: on the one hand, the interaction of high interdependence and high diversity can reduce team performance (Ely, 2004) and cause conflicts as it forces people with low levels of mutual trust and liking to interact. On the other hand, the decategorization hypothesis of task interdependence suggests that it might in fact ameliorate performance as frequent contact deconstructs stereotypes and prejudices and fosters fruitful collaboration (Chatman et al., 1998; Pettigrew, 1998). Henceforth, these contrary effects may have offset each other, accumulating in the observed null finding. Intergroup contact hypothesis (Allport, 1954; Pettigrew, 1998) offers four potential moderators of the positive versus negative effects of increased intergroup contact through task interdependence namely equality of status within the situation, shared goals, intergroup cooperation, and authority support. These potential moderators might be fruitful reference points to further investigate the influence of task interdependence on the effects of group diversity. In a similar vein, contrary to our expectations, psychological empowerment moderated neither the relationship between relationship conflict and strain (Hypothesis 4a), nor between task conflict and strain (Hypothesis 4b). The lack thereof may be due to ambivalent effects of empowerment (Spreitzer, 2008). While empowerment has generally been shown to bring about various benefits, it bears conceptual resemblance to the ambiguous role of challenge stressors discussed above. Particularly its meaning facet can enhance strain as people who show stronger commitment to their work tend to “take home” their successes and failures from work (Spreitzer et al., 1997).

### Implications

In sum, our study’s contribution to the literature is twofold. Theoretically, we successfully combined two influential models in organizational psychology, namely the social categorization model and the JD-R model. We empirically explored the ill-understood and largely neglected relationship between perceived team diversity and individual mental health outcomes. Moreover, our study elucidated the psychological underpinnings, revealing the critical role of task conflict as mediator of the association between perceived subgroups and strain (Hypothesis 2b).

In other words, perceived subgroups, as a consequence of social categorization processes, can hence be construed as a demand in the JD-R, eliciting strain through enhanced task conflict. The model can serve as a helpful conceptual starting point to guide future research at the crossroads of diversity studies and mental health.

Furthermore, offsetting shortcomings of prior, cross-sectional research, our study offers a longitudinal perspective. Thereby, we shed light on the neglected negative long-term impact of challenge stressors, such as task conflict on individual well-being. As this result is consistent with other longitudinal findings (de Lange et al., 2003; Hakanen et al., 2008; Sonnentag et al., 2010; Ford et al., 2014) but conflicts with the current appraisal of challenge and hindrance stressors, it might be worthwhile to critically reassess the basic mechanisms of the challenge-hindrance stressor framework over time.

Beyond that, the present results also have some practical implications. In line with the presented findings, practitioners would be well-advised to reconsider their approach to work engagement and psychological detachment, and consequently challenge and hindrance stressors. Whilst challenge stressors boost work outcomes, they risk inducing serious long-term strain and are thus potentially harmful to employees’ mental health. Many employers explicitly stress commitment to their workers’ well-being in their firm philosophies and mission statements. In order to live up to that, targeted interventions (Gao et al., 2017) and tailored stress management trainings (LePine et al., 2005; Widmer et al., 2012; Yao et al., 2015; Rispens and Demerouti, 2016) should be administered to empower employees to leverage the potential of team diversity and job challenges as much as possible, while minimizing the danger to their own mental health. That way organizations could create sustainable work environments, which ensure that professional gains do not come at the hidden cost of diminished employee well-being.

### Strengths and Limitations

Although subjectively perceived diversity has been shown to account for variance over and above objectively measured diversity (Harrison and Klein, 2007), it has been scarcely used in prior research (Thatcher and Patel, 2012). Acknowledging this shortcoming, the present study employed assessments of perceived subgroups, as the output, rather than input of social categorization processes at the workplace (Jehn and Bezrukova, 2010). Furthermore, previous work in diversity research has often relied on narrow, industry-specific samples, raising doubts about the applicability and validity of its findings to working environments in general (Mathieu et al., 2000; Hu and Linden, 2011). The present sample, consisting of a multitude of different teams from various professional and semi-professional contexts, ranging from academics and police forces to architects and musicians, marks an important step in overcoming this issue (Mumford et al., 2008). Of note, the JD-R model is very accommodating of highly diverse samples such as ours (Demerouti et al., 2001), which further attests to its suitability in this line of research. Similarly, the present findings underscore the importance and relevance of longitudinal studies in this strand of research, as critical outcomes may only unfold over time and might thus have been overlooked in the past, where the majority of studies drew from cross-sectional data.

At the same time, some limitations of the study should be noted as well. We employed self-reports for all measures, which might induce common method bias (Podsakoff et al., 2003, 2012). Moreover, in the absence of a published, well-established scale to measure perceived subgroups at the time of data collection, the authors jointly devised a questionnaire for the purposes of the present study. It should thus be highlighted that our newly-developed measure has not been properly validated despite yielding satisfactory reliability coefficients at both data collection time points (*α* = 0.78 at t_1_ and *α* = 0.85 at t_2_). Furthermore, predictor, mediator, and moderator variables were measured simultaneously, so that conclusions about their causal relationships are not possible.

### Future Research

The effects of diversity on mental health have been largely neglected, with the few existing studies mostly focusing on perceived discrimination, rather than perceived diversity *per se* (Pascoe and Smart Richman, 2009; Triana et al., 2015). Against this backdrop, scientists should delve more deeply into this important yet under-researched area, replicating and extending the findings of the present paper. Thereby, the fusion of the social categorization and the JD-R model may provide a theoretical grounding. Future work should aim to challenge, consolidate and further refine it through empirical testing.

Given our findings, future research should further investigate the promises and perils of both types of stressors, especially in the context of diverse work teams. As the analysis of performance variables was beyond the scope of the present work, these outcomes should be explored in coming studies.

For a variety of reasons (e.g., globalization, migration, demographic change, improved gender equality, and management strategy), we can assume work team diversification to proceed. The current study suggest that this process entails not only chances, but also risks. To ensure the success of the necessary transformational processes, interventions to contain potential undesired side effects such as diversity or stress trainings or improvements in workplace design should be implemented.

### Conclusions

Being, to our knowledge, the first study to directly examine the impact of perceived diversity on mental health issues at the workplace, we conclude: first, combining the previously unrelated, powerful social categorization model and JD-R model may provide an encouraging theoretical framework to study these two timely topics. Second, the present work suggests that the link between perceived diversity and strain rests upon task conflict as mediator. Task conflict may take an even greater toll on employee well-being in diverse teams than in more homogeneous groups. Building upon the challenge-hindrance stressor framework (LePine et al., 2005), it appears that adequately managing, rather than one-sidedly promoting challenge stressors (e.g., task conflict) and its precursors seems key to maintain satisfying performance outcomes without jeopardizing individual well-being. HR-managers might therefore shift their focus to act even earlier by targeting the formation of subgroups itself.

Notwithstanding the preliminary nature of our findings, we have some confidence in these conclusions as they are based upon longitudinal data from a conveniently-sized sample, affording a high degree of external validity. Nevertheless, much work remains to be done in this emerging line of research and future work needs to consolidate and extend our findings.

## Data Availability Statement

The data as well as the analysis and result scripts for the structural equation models are available in the OSF (https://osf.io/d765c/).

## Ethics Statement

Ethical review and approval was not required for the study on human participants in accordance with the local legislation and institutional requirements. The patients/participants provided their written informed consent to participate in this study.

## Author Contributions

All authors contributed to the design, data collection, and manuscript preparation. Data analyses were conducted by NS and BM.

### Conflict of Interest

The authors declare that the research was conducted in the absence of any commercial or financial relationships that could be construed as a potential conflict of interest.

## Footnotes

^1^https://osf.io/d765c/

## Appendix

Original German items of the scale perceived subgroups and their English translation.

**Table:**

German item	Translation
Gruppenmitglieder in unserem Team, die sich ähnlicher sind, haben mehr Umgang miteinander.	Members of our team who are similar to each other interact more frequently.
Innerhalb meines Teams bilden sich oft dieselben Gesprächsgruppen.	In my team, the same groups of people discuss among themselves.
Innerhalb meines Teams sind verschiedene Teilgruppen entstanden, deren Mitglieder sich gut verstehen.	Within my team, different subgroups have emerged whose members get along very well.

The respondents had to rate these items on a 7-point Likert scale from −3 = trifft überhaupt nicht zu/strongly disagree to 3 = trifft voll zu/strongly agree.
